# Congenital portosystemic shunt occlusion with an Amplatzer PFO occlusion device: a case report

**DOI:** 10.1186/s42155-021-00204-y

**Published:** 2021-01-11

**Authors:** Michelle M. Shnayder, Mario Dervishi, Alexandria Jo, Benjamin Pomerantz

**Affiliations:** 1 Department of Radiology; Division of Vascular and Interventional Radiology, University of Michigan Health System, 1500 E. Medical Center Dr, B1D502, 48109-5030 Ann Arbor, MI USA; 2 American University of the Caribbean School of Medicine, Cupecoy, St. Maarten

**Keywords:** Congenital portosystemic shunt, Extrahepatic portosystemic shunt, PFO occlusion device

## Abstract

**Background:**

Congenital portosystemic shunts are embryological malformations in which portal venous flow is diverted to the systemic circulation. High morbidity and mortality are seen in patients with concurrent hepatic encephalopathy, hepatopulmonary syndrome, and pulmonary hypertension. Endovascular therapy, in the correct patient population, offers a less invasive method of treatment with rapid relief of symptoms.

**Case presentation:**

In this report, we discuss the treatment of a two-year-old male with abnormal chorea-like movements, altered mental status, anisocoria and hyperammonemia diagnosed with an intrahepatic congenital portosystemic shunt between the inferior vena cava and right portal vein. Given the patient’s amenable anatomy and shunt type, embolization was performed with an 18 mm Amplatzer patent foramen ovale occlusion device.

**Conclusions:**

Portosystemic shunts are a rare congenital abnormality without universal treatment guidelines. An Amplatzer PFO occlusion device can provide a novel method of shunt closure given appropriate shunt type, size and anatomy.

## Introduction

Congenital Portosystemic Shunts (CPSS) are rare developmental malformations that occur in 1 in 50,000 births. [1] Some patients remain asymptomatic and the presence of an aberrant malformation is incidentally identified on imaging. However, a subset of patients become symptomatic at an early age and have increased morbidity. Common complications of CPSS include hyperammonemia, cholestasis, liver tumors, pulmonary hypertension or hepatopulmonary syndrome. [2]

Treatment options for symptomatic shunts include endovascular occlusion, surgical ligation, liver resection, and transplantation; however, endovascular occlusion has largely become the preferred less-invasive method of treating certain CPSS. Within the toolbox of endovascular occlusion options, vascular plugs and coils are frequently used with few case reports describing the use of Patent Foramen Ovale (PFO) Occlusion Devices (OD). [3, 4] Standard treatment guidelines for CPSS are not yet developed due to the rarity of this condition. Our report highlights the side-to-side closure of a CPSS with an Amplatzer PFO OD—an off-label use of this device.

## Case report

**Fig. 1:**
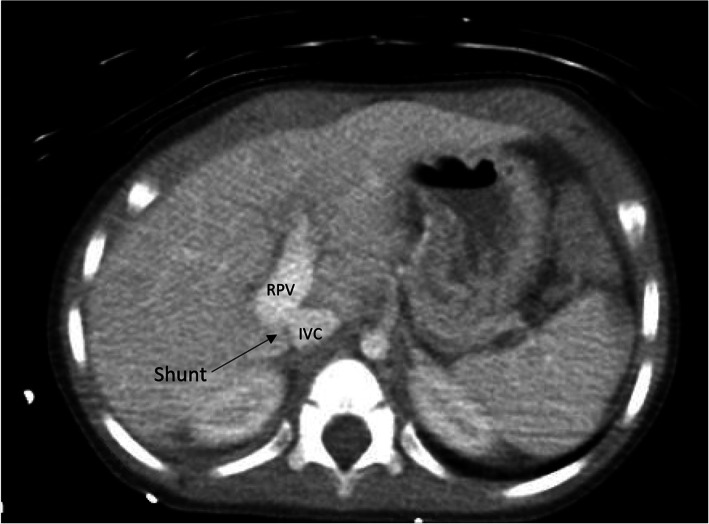
Pre-procedure imaging: Axial CT image demonstrating a portosystemic shunt (black arrow) between the right portal vein (RPV) and inferior vena cava (IVC) (labeled)

A two-year-old male with history of Trisomy 21, atrial septal defect per echocardiogram, and autism spectrum disorder presented to the emergency department with abnormal chorea-like movements, altered mental status, anisocoria. Laboratory findings showed hyperammonemia (maximum level of 256 mg/dL). The patient’s brain computed tomography (CT) and magnetic resonance imaging (MRI) were unremarkable. Doppler ultrasound of the abdomen demonstrated an intrahepatic shunt between the right portal vein (PV) and inferior vena cava (IVC). Hepatopetal intrahepatic portal venous flow was present in diminutive PV branches. Further evaluation with contrast enhanced CT of the abdomen confirmed the presence of a CPSS. (Fig. 1) The interventional radiology service was consulted for further evaluation. Treatment options were discussed with the cardiology team, and an Amplatzer PFO OD was deemed appropriate for the patient’s anatomy.

**Fig. 2:**
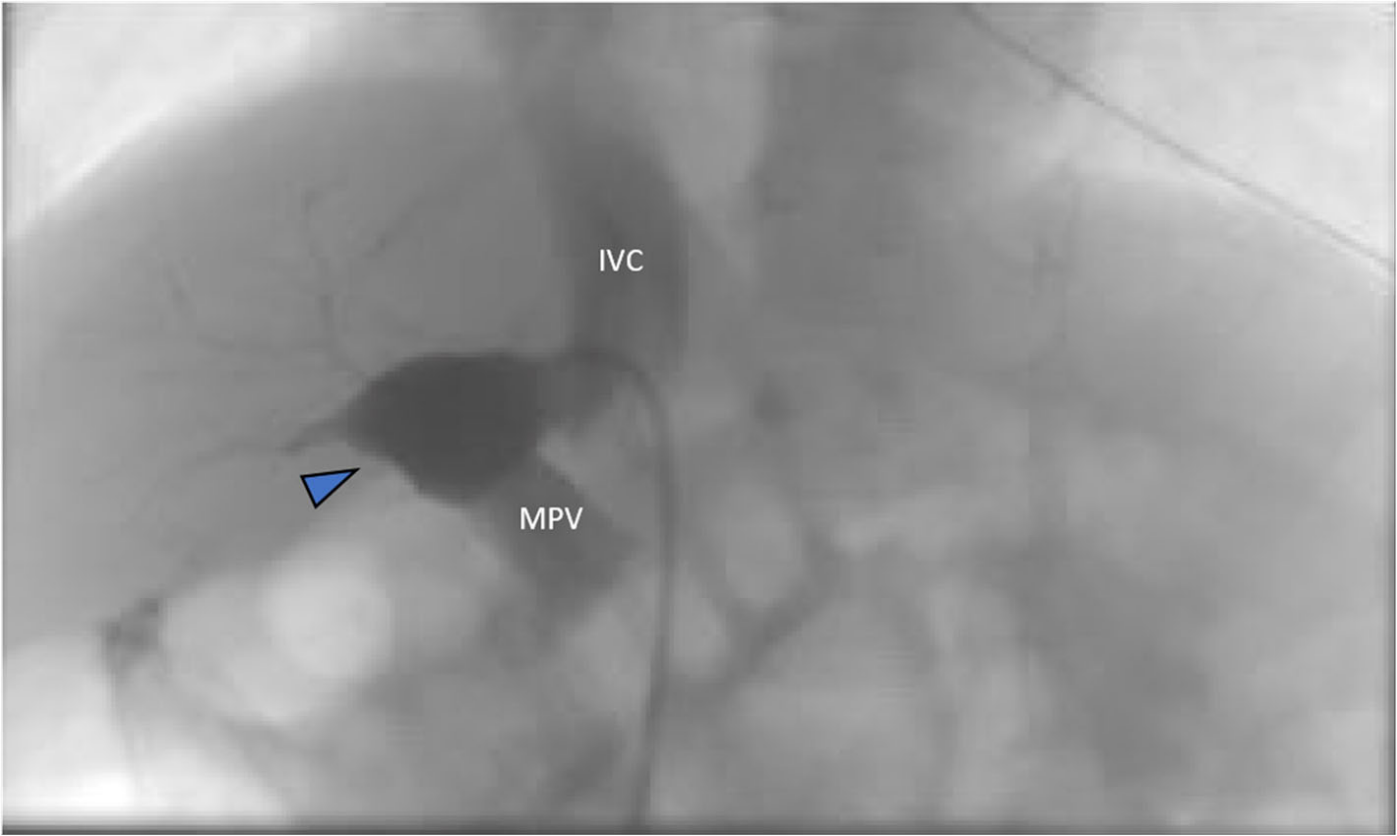
Fluoroscopic evidence of CPSS. Contrast injection upon cannulation of fistulous connection between IVC and right PV branch (blue arrowhead) with a 7F sheath. Contrast is seen flowing into the MPV (labeled) and superior portion of the IVC (labeled)

**Fig. 3:**
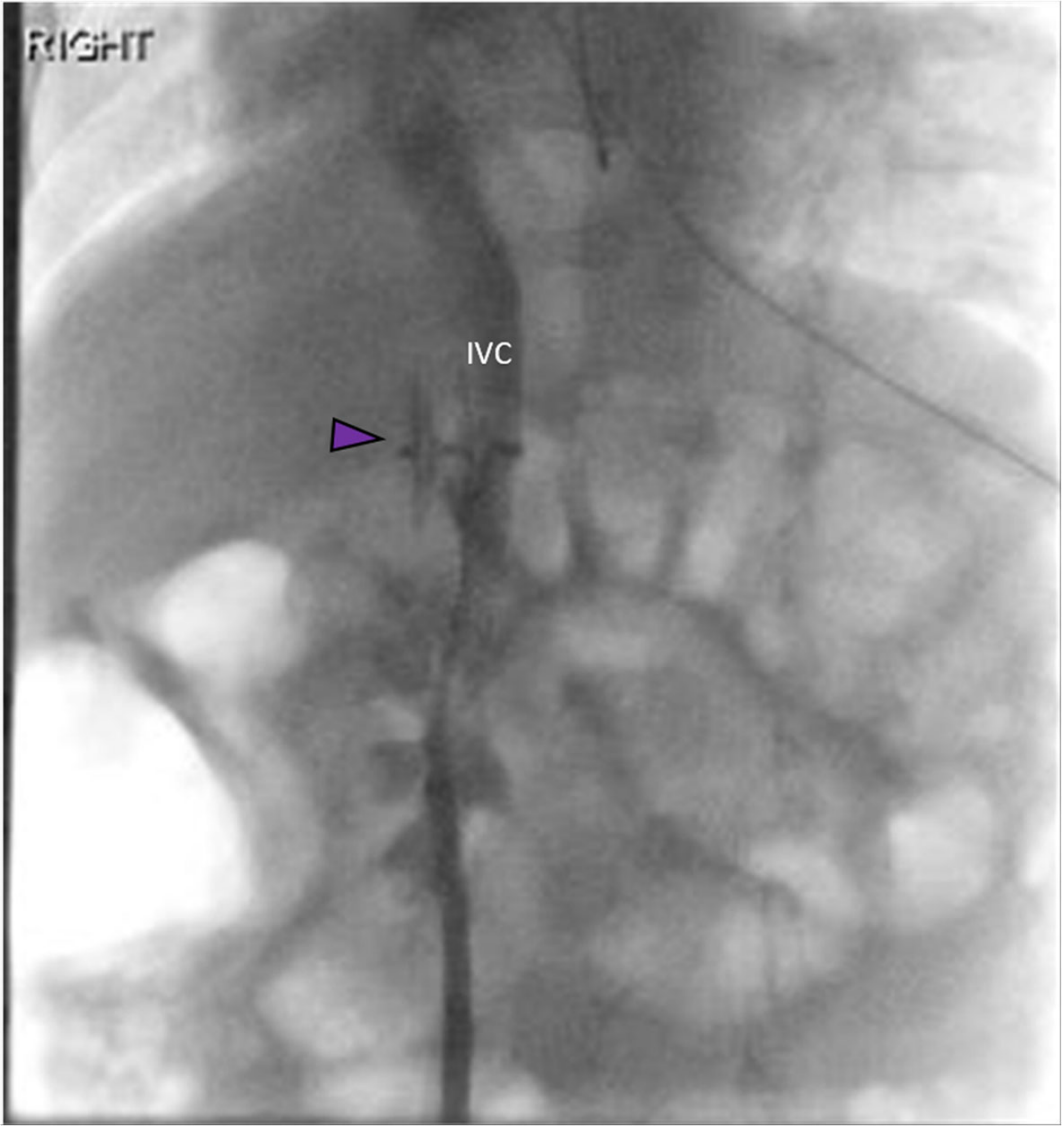
Occlusion of CPSS with Amplatzer PFO OD: Fluoroscopic placement of an Amplatzer PFO OD into the CPSS (purple arrowhead). Unidirectional flow of contrast is seen through the IVC (labeled) with absence of flow through the PV

**Fig. 4:**
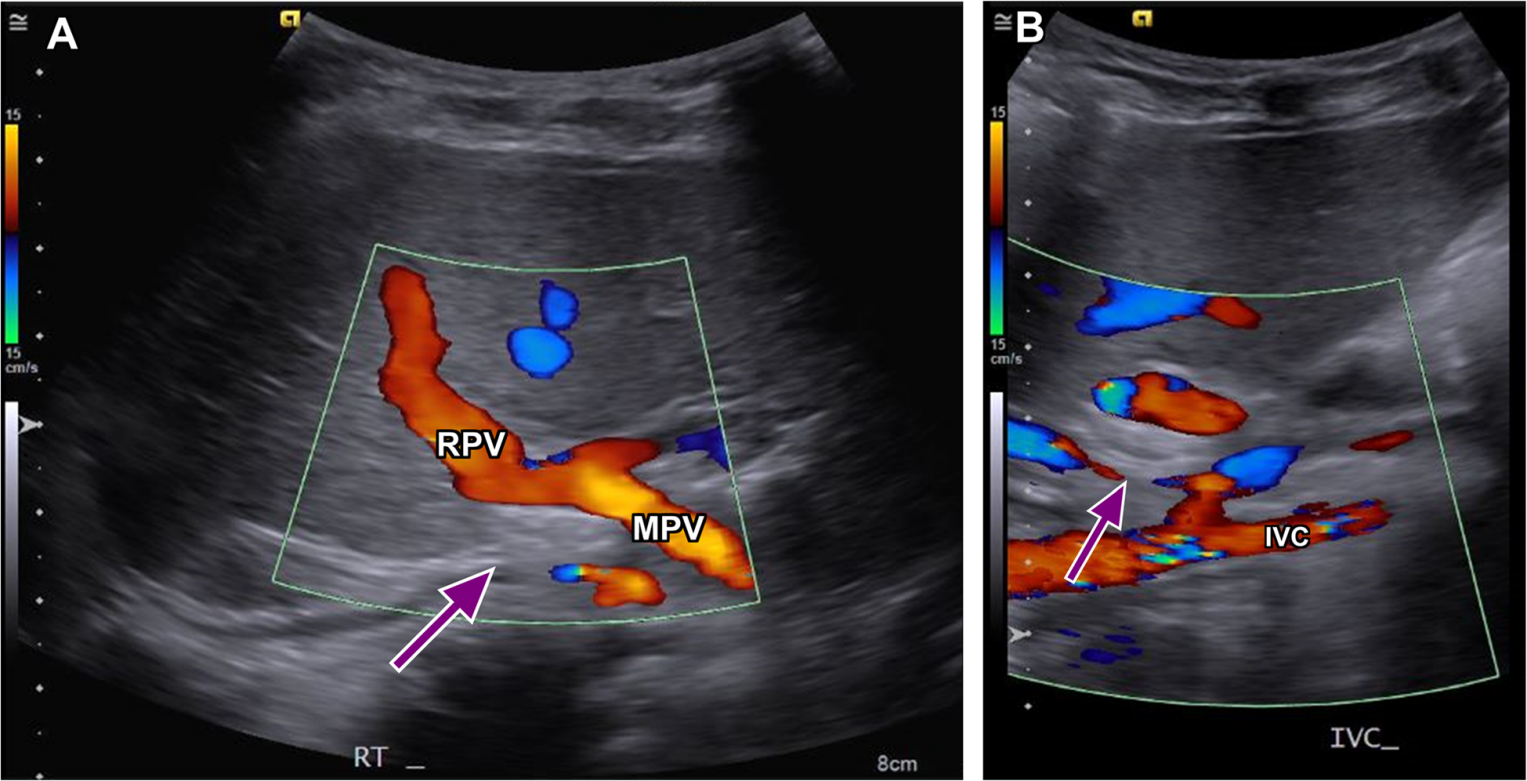
Post-procedure imaging: Doppler ultrasound images of the liver at one-month follow-up. **a** The Amplatzer PFO OD (purple arrow) is seen within the expected location of the previously seen CPSS with patent hepatopetal flow of the main portal vein (MPV) (labeled) and RPV (labeled). **b** The IVC (labeled) is patent

Right femoral vein access was obtained, and a venogram of the IVC confirmed a large side-to-side shunt from the anterior right lateral aspect of the IVC to a branch of the right PV measuring approximately 14 mm in diameter. (Fig. 2) The shunt was circumferentially surrounded by hepatic parenchyma with no additional side-branches. Due to the patient’s acute condition and the high likelihood of placing the occlusion device regardless of measurements, hepatic pressures were not obtained. Through a 7 French sheath, an 18 mm x 18 mm Amplatzer PFO OD (St. Jude Medical, Inc., Saint Paul, MN) was deployed across the shunt with the distal portion of the plug being deployed within the right PV branch. The sheath and plug were then retracted until the circumferential hepatic parenchymal flap resisted further traction. The second portion of the plug was subsequently deployed across the shunt and the device was detached. (Fig. 3) Post-occlusion venography showed successful shunt embolization. (Fig. 3) There were no immediate complications. Given mild bleeding from the right femoral puncture site due to patient movement, short-term anticoagulation was not prescribed. On post-procedure day one, the patient’s ammonia levels decreased to 55 mg/dL. He was discharged on day two with normal ammonia levels and baseline mental status. On one-month follow-up, a doppler hepatic ultrasound was obtained demonstrating persistent occlusion of the CPSS with patent hepatopetal PV flow and a patent IVC. (Fig. 4) At one-year follow-up, the patient was unable to comply with ordered laboratory and ultrasound studies due to significant apprehension and need for sedation. Given the patient’s excellent clinical status, ammonia testing and follow-up ultrasound was deferred. The patient’s mother reports dramatic improvement in his learning skills and energy levels greater than one-year post-procedure. No additional surgical or endovascular interventions have been required.

## Discussion/Conclusion

CPSS are rare intrahepatic or extrahepatic vascular malformations redirecting blood flow from the portal circulation to the systemic circulation, bypassing the liver. [2] Extrahepatic portosystemic shunts (EPSS) or “Abernethy malformations” have aberrant connections between the porto-mesenteric vasculature before PV branching and a systemic vein. [5] Type 1 EPSS have no portal venous perfusion of the liver while Type 2 EPSS have partial portal perfusion of the liver. Intrahepatic shunts, are classified based on the abnormal connections between intrahepatic branches of the portal vein and the hepatic veins or IVC. [5, 6] There are four subtypes which include: a single large vessel connecting the right portal vein to the IVC (type 1), localized peripheral connections between peripheral PV branches and the hepatic veins in a single hepatic segment (type 2), an aneurysmal connection between a peripheral PV and hepatic vein (type 3), and localized peripheral connections between peripheral PV branches and the hepatic veins in bilateral hepatic lobes (type 4). [5, 7] Our patient was classified as a type 1, intrahepatic CPSS.

Doppler ultrasound is the main mode of diagnosis of CPSS. [3] Pre-procedural CT angiography and magnetic resonance angiography are often used to further evaluate complex anatomy. Shunt type, location, degree of function, patient age, severity of symptoms, and complication risks are several considerations when planning treatment. [8] To date, no standard treatment options exist. Given the variability of treatment options, this case study seeks to describe when endovascular approach with an Amplatzer PFO OD is appropriate and how it can successfully be utilized to treat a large type 1 intrahepatic CPSS.

Asymptomatic patients diagnosed with an incidental finding of intrahepatic shunt prenatally or in early infancy should be monitored for a year before definitive intervention, as many intrahepatic CPPS involute by this time. [9] Symptomatic patients, such as our patient, require immediate treatment to avoid complications associated with encephalopathy and liver dysfunction. [8, 10] Shunt type also determines treatment approach. Many intrahepatic CPSS can be treated with endovascular occlusion or surgical ligation given the presence of other hepatic PV perfusion. For patients with a type 1 EPSS, liver transplantation is required for definitive treatment, while for type 2 EPSS, embolization remains a non-invasive treatment option. [5]

Careful evaluation of the shunt anatomy helps to determine the appropriate embolic agent for treatment. Considering our patient’s large diameter shunt and high flow, endovascular coils and detachable balloons were considered high risk for migration. Although vascular plugs have frequently been used for treatment of CPSS, few case reports describe the use of the Amplatzer PFO OD. [3, 11, 12] Due to near parallel anatomical alignment between the right PV and IVC and as there was a circumferential hepatic parenchymal flap similar to a PFO, an Amplatzer PFO OD was determined to be a reasonable treatment option. The device’s double disc design enabled placement of one disk in the right PV and the second in the IVC as it flanked the flap. Careful deployment of the device between the two vessels was ensured to avoid fatal outcomes by occlusion of either the right PV or IVC. This device was also chosen in part due to its ease of relocation and retrievability in case of improper positioning. Suboptimal anatomy including an end-to-side shunt or diminutive IVC and portal venous branches would preclude the use of an Amplatzer PFO OD.

Traditionally, surgical ligation has been the main therapeutic treatment for shunts with high flow rates; however, when possible, percutaneous approaches can offer a less invasive and rapid correction of symptoms. [8] An Amplatzer PFO OD should be considered in endovascular treatment of a large CPPS when the anatomy is amenable.

## Data Availability

Not applicable.
